# Confronting Tradeoffs Between Agricultural Ecosystem Services and Adaptation to Climate Change in Mali

**DOI:** 10.1016/j.ecolecon.2018.04.003

**Published:** 2018-08

**Authors:** Kurt B. Waldman, Robert B. Richardson

**Affiliations:** a Department of Geography, Indiana University, 513 N. Park Ave., Bloomington, IN 47408, USA; b Department of Community Sustainability, Michigan State University, 480 Wilson Rd., East Lansing, MI 48824, USA

**Keywords:** Agricultural ecosystem services, Choice experiments, Climate change adaptation, Gender, Mali, Sorghum

## Abstract

Changing climatic conditions present new challenges for agricultural development in sub-Saharan Africa. Sorghum has proven to be an adaptable and resilient crop despite limited funding for crop development. Recent breeding efforts target hybrid and perennial technologies that may facilitate adaptation to climate change. Advantages of perennial crops over their annual counterparts include improved soil quality and water conservation and reduced inputs and labor requirements. In contrast, hybrid crops are often bred for improved grain yield and earlier maturation to avoid variable conditions. We use discrete choice experiments to model adoption of sorghum as a function of attributes that differ between these technologies and traditional varieties in Mali. Overall, the main perceived advantage of perennial crops is agricultural ecosystem services such as soil improvement, while adoption of hybrid crops is hampered by the inability to reuse seed. Women farmers are less concerned about higher labor requirements associated with perennial crops and the ability to reuse hybrids seeds than male farmers. Farmers prefer traditional sorghum to perennial sorghum and are indifferent between traditional and hybrid sorghum. These findings have important policy implications for understanding tradeoffs that are central to farmer decision making when it comes to breeding technologies for climate adaptation.

## Highlights

• Breeding perennial sorghum varieties may contribute to ecosystem services improvement.
• Discrete choice experiments are used to model adoption of perennial and hybrid sorghum in Mali.
• Preference for perennial sorghum is related to potential soil improvement.
• Inability to recycle seed is an overwhelming deterrent for hybrid sorghum adoption.
• Farmers prefer traditional over perennial sorghum and are divided about hybrid sorghum.

## 1 Introduction

Ensuring access to nutritious and environmentally sustainable food to all people at all times is one of the greatest challenges currently facing global society (Hall et al. 2017). Climate change is projected to impact food security in the developing world, especially in countries already facing chronic hunger and malnutrition (Lobell et al. 2008). Climate change is expected to have a direct impact on food production as changes to both temperature and rainfall patterns affect crop yields, water availability, pests and diseases, and livestock health, and smallholder farming systems in Africa (Hall et al. 2017). Countries in sub-Saharan Africa are predicted to be worst affected due to already high temperatures, high dependency on rain-fed agriculture, and economic fragility. The problem is particularly acute in West Africa, where the dual forces of population growth and climate change are likely to exacerbate the persistent challenge of food insecurity. West Africa is also subject to significant rainfall variability and drought occurrences, and this is expected to increase as climate change impacts the region (Mason et al. 2015).

Sorghum is among a limited number of crops that have the resiliency to adapt to changing climate change conditions, particularly increasing drought, soil salinity and higher temperatures (ICRISAT, 2015). Despite these benefits, agricultural policies concerning subsidies and crop improvement have been inconsistent and often do not target sorghum. In the past two decades, domestic production has increased at an average rate of about 1% for sorghum, while maize has increased at an average rate of about 7% (Kelly et al. 2012), despite a steady increase in sorghum grain yield in West Africa since the 1970s (ICRISAT, 2015). Predicted increases in climate variability require strategies that improve the genetic heterogeneity of sorghum traits and improve the capacity for adaptation to increased variability (Haussmann et al. 2012).

Farmers require a diversity of crop options that can utilize different spatial niches of nutrients and water resources, and have different mechanisms for responding to annual variation in precipitation (Altieri 2002; Hall et al. 2017). Diversification is one strategy that allows farmers to spread short-term risk and create a more resilient farm system within the context of increasing climatic variation. Developing crop varieties that are resilient to variable climate conditions can improve the diversity and resilience of these systems (Morton 2007). Farmers in West Africa experience both long-term and year-to-year variability in rainfall and access to diverse crop types can improve their resilience (Mason et al. 2015).

Hybrid crops and perennial crops represent two radically different technologies which each have the potential to improve farmers' ability to adapt to variable climatic conditions. Through hybridization, desirable traits from diverse germplasm may be crossed with traditional landraces in order to create more productive varieties and varieties that combine useful traits, such as disease resistance, with regionally adapted and accepted plant morphology. Development of early maturing hybrid varieties of crops is a common way to reduce exposure to climate variability by reducing the amount of time a crop is in the field.

Cultivation of perennial crops on the other hand, is an investment in the long-term resilience of cropping systems. Perennials potentially fill an important niche for farming households and may have numerous ecological and agronomic advantages over their annual counterparts (Glover et al. 2012). While perennial crops often have drawbacks such as lower yields, many of the ecosystem services associated with the perennial nature of the crop make it more resilient and potentially an important component of climate change adaptation, particularly in water scarce areas.

There has been little research to date on preferences for crop duration—or more specifically household-level tradeoffs and preferences for long duration or perennial crops versus short duration, early maturing hybrids—and the prospects for using these technologies for climate adaptation. The literature on the economics of and preferences for perennial crop production in Africa is thin, largely because the development of perennial crops is in a nascent stage. Most of the literature focuses on plant physiology or developing breeding lines that have the capacity to regrow post-harvest and produce yield over successive years (Hayes et al. 2012; Kane et al. 2016). Breeding programs in Africa target a variety of crops from maize and beans to lesser-known or more traditional crops like sorghum and millet but little attention has been paid to the tradeoffs involved with perennial version of these crops.

There has also been relatively little attention to the gender implications of crop preferences. Many rural African smallholder societies are patriarchal and men make most of the agricultural decisions for the household, even though women are often the providers of food for the family. However, in polygamous households, common in Mali, women often manage land and make agricultural decisions independent of their husbands. In addition to examining the differences in tradeoffs embodied by perennial and hybrid crops, we specifically consider the variation in preferences among attributes of hybrid and perennial varieties of sorghum between male and female farmers.

## 2 Background

Depending on the agroecological region, sorghum, millet, and maize are the primary staple crops in Mali. Sorghum is widely cultivated across the Sudan Savannah and covers a range of agroecological zones characterized by a gradation in rainfall from the edge of the Sahara Desert to the fertile Niger River Delta. Development efforts led by the Malian government have been aimed at improving staple crop yields since the drought years that occurred in the 1970s and 1980s. Initially these efforts involved breeding with foreign genetic material but more recently has been focused on Guinea races that are traditional West African sorghum varieties (Rattunde et al. 2013).

Adaptation requirements for new sorghum varieties are specific to the agroecological zone, and no single variety predominates across all of the sorghum growing areas in Mali (Bazile et al. 2008). Yapi et al. (2000) found that farmers prefer the local sorghum landraces in Mali more than varieties derived from foreign genetic material. Local breeding programs developed both pure Guinea-race hybrids as well as Guinea-caudatum hybrid varieties in response.^1^ The Guinea race of sorghum has a relatively broad geographic distribution, and research has shown that it comprises more genetic diversity than other races (Folkertsma et al. 2005). Given the wide range of agroecological zones in Mali and the highly variable climatic conditions there is a need for a diversity of plant types.

The backbone of the green revolution in Asia was the development of high yielding hybrid varieties of crops that respond well to fertilizer (Eicher 1995). Hybridized versions of crops can address specific problems that plague farmers by breeding in pest or disease resistance, drought tolerance, resistance to abiotic stress that may problematic in a given region. Hybrid breeding, particularly with maize in Africa, has focused on improving low yields, but also more recently on developing shorter duration varieties that are more suitable for shortened growing seasons in many regions. There are drawbacks with hybrid crops, notably that hybridized seed tends to be more expensive and can only be planted once before hybrid vigor is lost. Hybrid crops also tend to require greater fertilizer inputs than local varieties to achieve their yield benefits. However, research has shown that despite the higher inputs costs, hybrid crops can return more per acre because of the greater yield advantage, which is true of hybrid sorghum in Mali (Smale et al., 2016).

Perennial lines of sorghum have only recently been tested in Mali and are at this point still hypothetical to farmers. The primary tradeoff with perennial crops is yield reduction since most perennial crops yield less than their annual counterparts (Pimentel et al. 2012). For this reason, efforts to develop perennial wheat in developed countries have focused on improved grain yield, and to a lesser extent grain quality (Cox et al. 2006; Jaikumar et al. 2012; Hayes et al. 2012). There are numerous advantages to perennial crops such as lower seed inputs since they require reseeding every three to five years as opposed to every year like annual crops (Bell et al. 2008). Less tillage also translates into less soil disturbance and erosion. As a result, the farm labor costs, energy usage, and technological inputs required for yearly tillage will significantly decrease in perennial crop systems (Pimentel et al. 2012). Additionally, the large root mass helps retain soil, prevent future erosion, sequester more carbon, and hold more soil water (Glover et al. 2010). Perennial cropping systems also have natural mechanisms that make them more resilient to pests (Van Mele and Chien 2004; Cox et al. 2006). Perennial systems have more soil fauna diversity and natural below ground processes since they have more year-round vegetation (Culman et al. 2013). Perennials are also more efficient than annual crops at absorbing nutrients, so fertilizer requirements are lower and nutrient leaching is reduced.

Perennial crops present attributes and management requirements that are very different from the attributes of the hybrid varieties more commonly found in breeding programs. In many ways, perennial crops are the antithesis of the typical hybrid crop variety, which is often input-intensive, early maturing, and high yielding. Perennial crops are not necessarily high yielding, they have lower labor requirements and are longer duration, but they may be more amenable to the low-input, low-output farm management style in Africa. Perennials are adaptable to climate change through extensive root growth that permits greater access to water and nutrients during dry spells, and they are thus often associated with improved agricultural ecosystem services. Hybrid varieties on the other hand tend to be bred to fit into a new and different cropping system that is characterized by more variable rain or a shorter growing season. Early maturing hybrids can avoid changing climatic conditions and variable rainfall by through crossbreeding with varieties that are short duration. Hybrids can also be bred for tolerance to temporal midseason drought and dry spells, and are often associated with adaptation to climate change.

Hybrid and perennial crops present different tradeoffs to farmers and this paper examines farmers' preferences for these different cropping systems and the associated attributes of each using choice experiments. We employ two sets of choice experiments to look at farmers' preferences for attributes of perennial and hybrid crops using the case of sorghum in Mali. We also chose to design the study with a gender focus since the structure of households in Mali is such that most male and female farmers manage agricultural land independently and are likely have independent preferences for crop attributes. We are most interested in the heterogeneity in preferences, with specific attention paid to differences by gender of the respondent so that we are able to characterize the relationship between crop duration and gender.

## 3 Methods

### 3.1 Study area and data

**Fig. 1:**
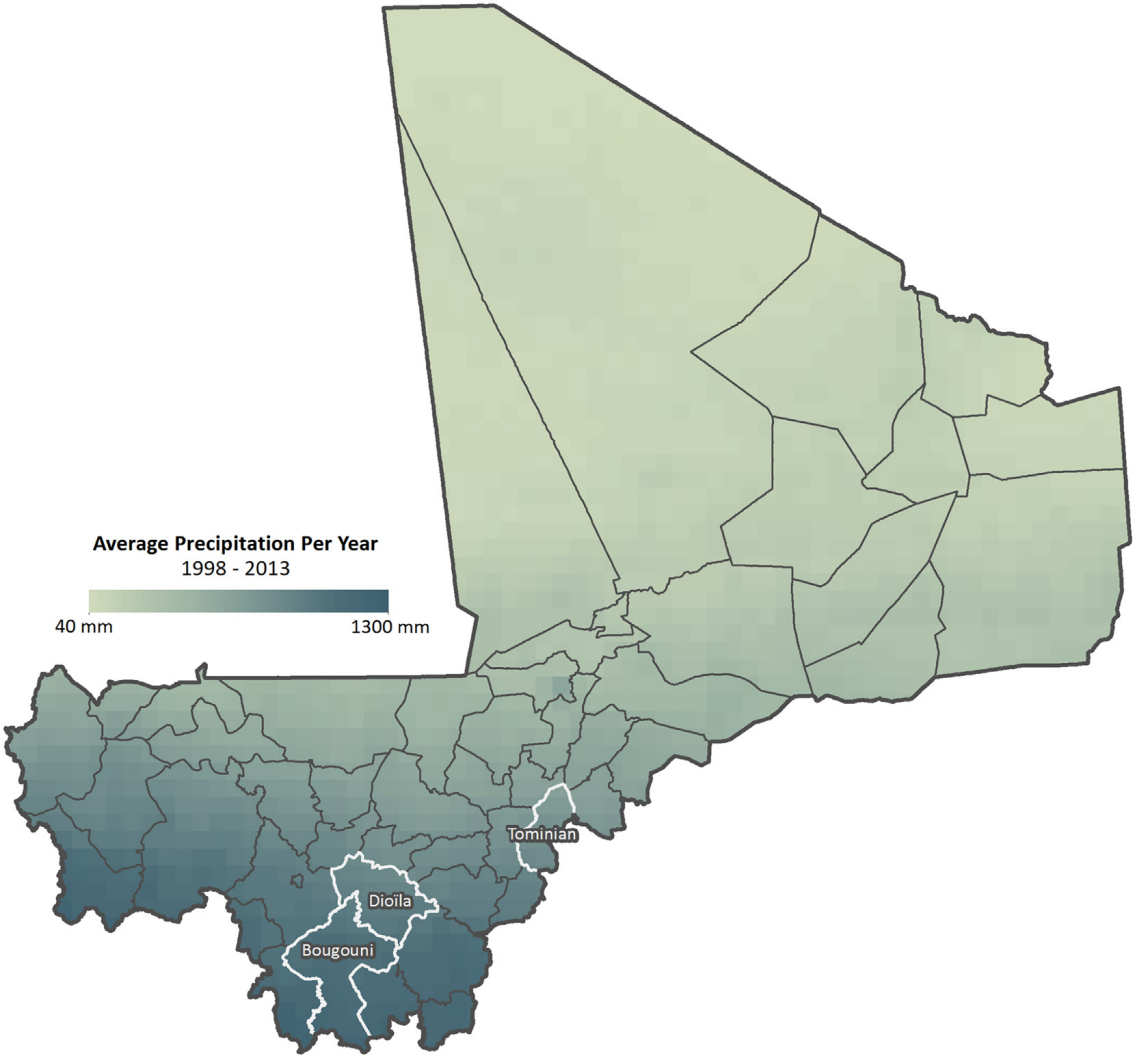
Precipitation map of Mali with research locations highlighted.

Fig. 1 depicts the rainfall gradient and the study sites in Mali. The gradient runs from the southern border with the Ivory Coast, where the highest rainfall occurs (about 1400 mm annually) northeast to the border of the Sahara desert, where rainfall is too low to support crop cultivation (below 400 mm of rainfall per year). This rainfall gradient also extends across the rest of West Africa. There are four main agroecological zones in Mali of which the Sahelian zone and Saharan Zone are too dry to cultivate. Sorghum is cultivated in the southern half of the country in the Subhumid Zone and the Sudanian Savannah, which comprise 6% and 12% of land respectively. In the Sudanian Savannah, the soils are relatively heavy and sorghum is the dominant cereal crop, often rotated with cotton, maize, groundnuts or cowpeas.

We use a spatially explicit clustered multistage sampling approach to capture variation along the rainfall gradient. Out of the 8 regions in Mali, we select three regions where annual sorghum is cultivated under low, medium, and high rainfall; Sikasso, Koulikoro, and Segou, respectively. Within each region we select one Cercle, which is the second level administrative unit. The selected Cercles were Bougouni, Sikasso; Dioila, Koulikoro; and Tominian, Segou (highlighted in Fig. 1) and we randomly choose six villages from a list of all villages within each Cercle. Prior to visiting households we received permission from village elders to interview 20 people in each village (approximately 10 men and 10 women) and local guides brought enumerators to houses at an increasing distance from the center of the village. We have a total of 318 complete observations after discarding observations from two villages in Bougoni where there were some problems with initial survey enumeration quality.

The Cercle of Bougoni is at an elevation of 344 m, covers 20,028 km^2^ and according to the 2009 Census, has a total population of 459,509 and a population density of 23/km^2^. The average elevation of Dioïla Cercle is 331 m and it covers a total area of 12,794 km^2^ with a population of 249,403 and a population density of 19/km^2^. Tominian Cercle sits at an average elevation of 301 m, covers a total area of 6573 km^2^ with a total population of 219,853 and a population density of 33/km^2^. Annual rainfall ranges between 450 and 700 mm in Segou region and the Tomininan district is located at North in the driest part of the region. In Koulikoro region, Dioïla has a rainfall pattern which ranges between 800 and 900 mm per year. While in the Sikasso region, often referred to as the “bread basket” of Mali, the annual rainfall varies between 800 mm in the North to 1200 mm in the South-West. Bougouni is located in the wettest part of the region.

Sikasso and Koulikoro regions are the principal sorghum-producing regions, with 31% and 22% of national production respectively (Kelly et al. 2015). These areas have been priority target areas for sorghum breeding, especially for hybrid development in Mali since sorghum is the primary staple in much of this zone (ICRISAT, 2015). Rattunde et al. (2013) found average yield advantages of 380 kg/ha to 660 kg/ha with hybrids relative to an adapted landrace.

### 3.2 Choice experiments

We use choice experiments to study farmers' preferences for the attributes of hybrid and perennial sorghum crops. Choice experiments measure stated preferences of participants as opposed to revealed preferences that come from observed retail transactions, yet perform similarly to market price analysis (Scarpa et al. 2003). Choice modeling is a method used to estimate the marginal value of various attributes of a good and is especially useful when observed transactions of a good do not occur (Train 2009). Choice experiments are a flexible tool useful for understanding hypothetical choices and demand for new agricultural products or technologies (Useche et al. 2009; Useche et al. 2013). To minimize hypothetical bias, increase comprehension of the choice experiment, and reduce the cognitive burden of the exercise, the choice sets were illustrated and presented to farmers on laminated cards.

Choice experiments are widely used in the agricultural and environmental economics literature and are increasingly common for understanding preferences in a development context (Bennett and Birol, 2010). They can be used to understand breeding traits in different production systems (Roessler et al. 2008), to estimate willingness to pay for a new agricultural technology (Birol et al., 2012), to preferences for cropping systems (Ortega et al. 2014), and investigate preferences for environmental adaptability and yield stability (Asrat et al. 2010). Numerous studies use choice experiments to evaluate preferences of developing country farmers concerning environmental or ecological tradeoffs involved in agricultural production. Choice experiments were used to estimate demand for perennial pigeon pea in Malawi (Waldman et al. 2017), and they have also been used to estimate Mexican farmers' preferences for biodiversity in their cropping systems (Birol et al. 2009). Choice experiments were used in the analysis of tradeoffs regarding pineapple production and environmental management in Costa Rica (Richardson et al. 2013), and to value incentives to households that communally manage natural resources (Pienaar et al. 2014).

Choice experiments have also been used to measure farmer adoption of improved staple crop technologies (Sánchez-Toledano et al., 2017a, Sánchez-Toledano et al., 2017b). We build on this literature by using choice experiments to understand preferences for an agricultural technology with strong ecological advantages that is a nascent stage of breeding development, and compare them with preferences for an existing technology, with an emphasis on tradeoffs between agricultural ecosystem services and adaptation to climate change.

#### 3.2.1 Modeling preferences for perenniality using choice experiments

We design two choice experiments, one examines preferences for hybrid sorghum and the other examines preferences for perennial sorghum. In both, we assume a farmer chooses between perennial or hybrid and annual sorghum, and we assume that farmers seek to maximize the utility derived from their cropping decision. We use random parameters logit (RPL) model that allows us to control for the standard deviation in the choice attributes. We are interested in exploring the heterogeneity among preferences of individuals as opposed to a latent class approach, which involves identification of groups of farmers that are heterogeneous across classes and homogenous within a group. We explicitly want to understand how individual preferences differ, particularly along gender lines. An RPL model with interaction effects between the choice attributes and the gender variable is more consistent with this aim than a model such as the covariance heterogeneity model which allows for scale to be a function of attributes.

We suppose that farmer *n* faces *K* alternatives contained in choice set *ψ* during situation *s*. We define an underlying latent variable *V*_*njs*_^∗^ that denotes the value function associated with farmer *n* choosing option *j* ∈ *ψ* during choice situation *s*. Farmer *n* will choose alternative *j* so long as *V*_*njs*_^∗^ > *V*_*nks*_^∗^ ∀ *k* ≠ *j*. Indirect utility *V*_*njs*_^∗^ is not directly observed; what is observed is the actual utility maximizing choice *V*_*njs*_, where

(1) Vnjs=10ifVnjs∗=maxVn1s∗Vn2s∗…VnKs∗Otherwise

And we can therefore write farmer *n*'s utility function as

(2) $$V_{\mathrm{njs}}^{*}=X_{\mathrm{njs}}^{\prime }\beta +\varepsilon_{\mathrm{njs}}$$

where *X*_*njs*_^′^ is a vector of characteristics of the cropping system for the *j*th alternative, *β* is a vector of preference parameters or vector of weights that map the attribute levels into utility, and *ε*_*njs*_ is a stochastic component of utility that is independently and identically distributed (iid) across individuals and alternative choices, and takes a predetermined (Gumbel or extreme value type I) distribution. This stochastic component of utility implies that predictions cannot be made with certainty and captures unobserved variations in preferences as well as errors in farmer's perceptions and optimization.

We estimate perennial and hybrid sorghum models in both preference space and willingness-to-pay (WTP) space. The coefficients on the attributes in the preference space model reflect the marginal utility of each attribute to an individual and the ratio of two marginal utilities is the marginal rate of substitution of one attribute for another. The advantage of estimating a model in WTP-space is that one can estimate the marginal rate of substitution directly (Scarpa et al. 2008). By specifying an attribute for comparison, one avoids the problem of comparing attributes from coefficients with different scales. Similar to Ortega et al. (2016), we estimate WTP for choice attributes in terms of the amount of crop yield an individual would be willing to substitute for each of the other attributes.

The most important tradeoffs and design attributes of the choice sets for annual, perennial, and hybrid sorghum production were estimated from a combination of literature review, informal discussion with breeders, and a series of focus groups with farmers in Mali in October 2014. We also consulted with local agronomists and experts in legume cultivation in Mali to design the parameters for the choice experiment. We identified the main attributes to be used in the choice experiments through informal interviews with farmers and consultation with agronomists and made a series of modeling assumptions based on these interviews.

Rather than present one choice as perennial and one as annual we decided to decompose the main attributes of a perennial crop in order to understand valuation of the attributes without the stigma that may be attached to other perennial crops or varieties of sorghum that are known to regenerate. This is consistent with Lancaster's theory of consumer behavior, which states that consumers are not seeking the goods themselves but the characteristics they embody (Lancaster 1966). Some farmers were aware of the concept of hybrid and perennial sorghum and we wanted to avoid associating their choices with their perceptions of these crops so we avoided labeling the choices as such. We model sorghum as a sole crop that is part of a crop rotation as opposed to intercropped, although various forms of intercrop are common in Mali.

The yield reduction from a perennial crop was identified as a key factor in farmers' decision-making processes so we include this as a “price variable” in the choice experiment. Given the prevalence and importance of sorghum in Mali, this attribute serves as a substitute for a cost or price variable when evaluating tradeoffs with the other attributes. This indirect measure is preferred over a direct monetary variable, as yield is a more intuitive measure of value to farmers given the subsistence nature of agriculture in the region (Birol et al. 2009). This is also consistent with the notion of staple grains such as sorghum serving as a form of currency for smallholders (Smale et al., 2016). We chose to frame sorghum yield as a percentage of an average year's harvest in order to avoid having to specify the plot size being harvested, or control for variation between villages and farms due to environmental factors.

Other factors identified as critical tradeoffs concerning annual versus perennial sorghum was the reduction in seed and labor requirements resulting from a single planting with multiple harvests. We also model the benefits of perennial sorghum, which theoretically include soil improvement from more extensive perennial root growth, and higher levels of biomass/forage, since less energy is required for root development. We assume that breeders can reach a point where perennial sorghum can reliably survive the dry season and regenerate but that on average the yield of perennial sorghum is lower than traditional sorghum. With hybrid sorghum, we assume less seed is required, the price of the seed is higher than traditional sorghum seed, and the seeds cannot be reused in subsequent years. Furthermore, we assume that there are multiple plant durations of hybrid sorghum, and the grain yield of hybrid sorghum is higher on average than traditional sorghum.

#### 3.2.2 Parameterization of the choice models

Smale et al. (2016) calculate the unit variable cost of traditional (annual) sorghum to be 125 CFA/kg producing an average of 950 kg/ha, improved sorghum to be 105 CFA/kg producing an average of 1500 kg/ha and hybrid sorghum the lowest at 65 CFA/kg or 2500 kg/ha.^2^ Based on these figures we model the choice between a traditional and perennial crop (or hybrid crop) as a reduction (or increase) in yield based roughly on the variation in current versus previous yield of traditional varieties and the percentage range observed for perennial or hybrid to traditional. The values of the parameters were wide enough to capture both the high and low ends of the spectrum.

Cropping system attributes used in the perennial choice experiment included seed requirements, labor requirements, forage quantity, soil improvement, and sorghum yield. Cropping system attributes and their levels are described below in Table 1. We modeled the variation in seed requirements as ranging from the same requirements as their current varieties to 15% and 30% reductions to capture the lower amounts of seeds required for perennial cultivation. We treated labor as a binary variable (low and high) since there is such wide variation in labor availability across farms and regions. Perennial crops tend to produce much higher levels of above-ground biomass since they have to devote less energy to root production after establishment, and so we have also modeled this variable as a binary choice to avoid misleading amounts, since we do not have accurate quantification of levels of biomass from perennial sorghum. Similarly, we modeled soil improvement as a binary variable since one of the often-stated benefits of perennial crops is long-term soil improvement from the additional carbon resulting from the larger root mass, although we do not have empirical evidence of the magnitude. Modeling attributes as binary that are continuous is a significant limitation of the study but in the absence of detailed empirical estimates this approach allows us to evaluate how important this attribute is to farmers.

**Table 1:** Attributes used in the perennial sorghum choice experiment.

Attribute	Levels	Description
Seed requirements	Average, −15%, −30%	Amount of seed required compared to current sorghum varieties
Labor requirements	Low, high	Amount of labor required with the sorghum crop/variety
Forage quantity	Low, high	Level of residue produced over the planting period
Soil improvement	Low, high	Extent that the soil quality is improved from a crop
Sorghum yield	Same, −20%, −40%, −60%	Percent of previous year sorghum grain yield

The main attributes identified as important in the decision to plant hybrid versus traditional sorghum varieties were the seed requirements, the plant duration, the ability to reuse the seeds, the cost of the seeds and the grain yield (see Table 2). We included two attributes of seeds that differ between traditional and hybrid varieties—the amount of seed required (hybrid seeding rates recommended by seed companies are roughly half that of local varieties) and the cost of the seed (approximately 600 kg for hybrid, 100 kg for traditional) (Smale et al., 2016).

**Table 2:** Attributes used in the hybrid sorghum choice experiment.

Attribute	Levels	Description
Seed requirements	5, 10 kg/ha	Amount of seed required compared to current sorghum varieties
Plant duration	Early, Medium, Late	The days to maturity of the plant (early is about 4 months while late is >6 months)
Ability to reuse seeds	No, Yes	Whether the seeds can be harvested and reused in the following season
Cost of seed	100 CFA, 600 CFA	The cost of 1 kg of the seed
Sorghum yield	120%, 140%, 160%, 180%	Percent of previous year sorghum grain yield

**Fig. 2:**
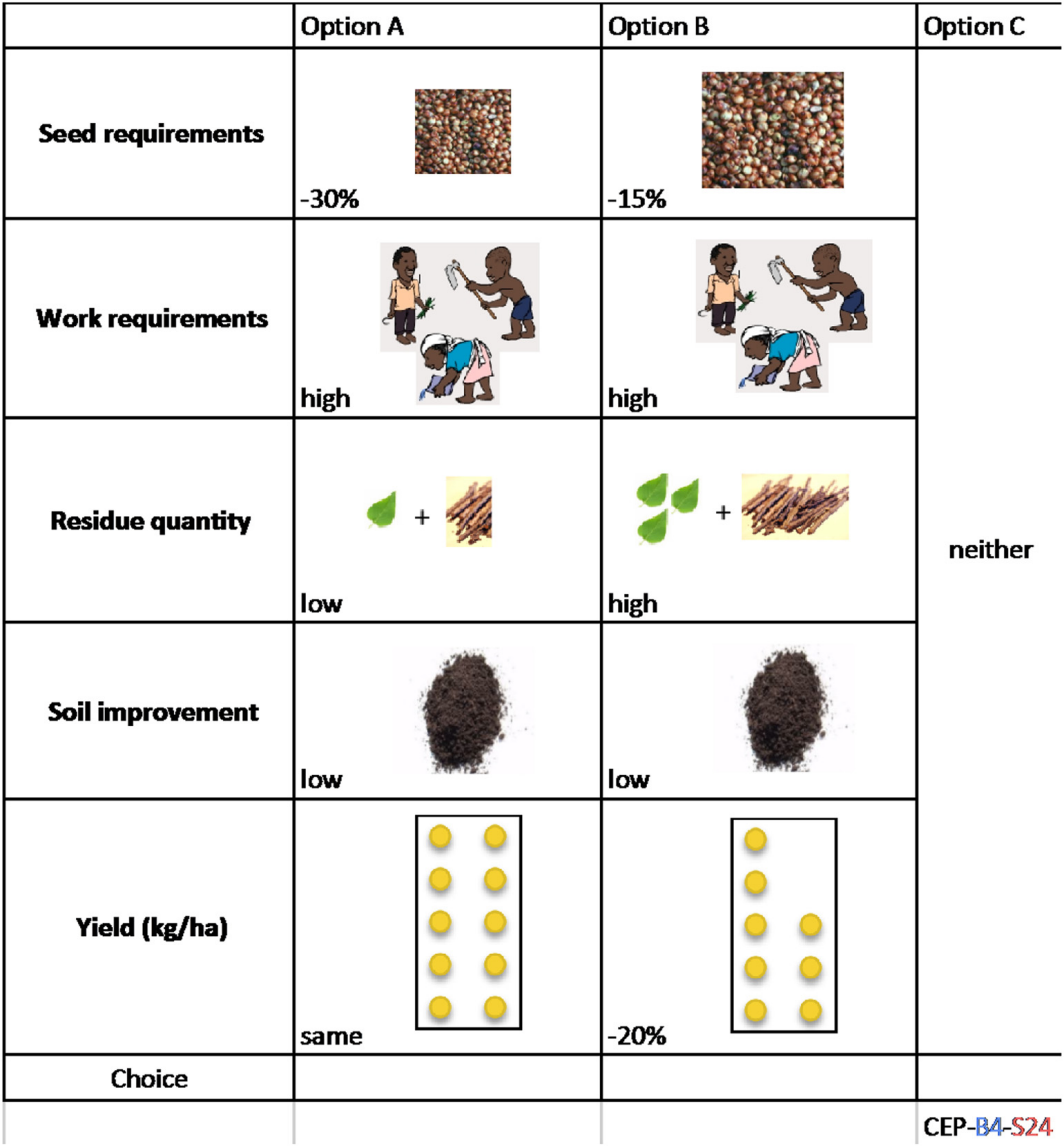
Sample choice set for perennial sorghum experiment.

We created an orthogonal experimental design using these attributes and levels using the software Ngene 5.0. The experiment was divided into 6 blocks with 4 choice sets in each block. An option to opt out or select “neither” choice was also presented each time, labeled option C. We created illustrated booklets of the choice sets in each of the 6 blocks. A sample choice set is presented in Fig. 2.

## 4 Results

### 4.1 Descriptive statistics

We present basic summary statistics for farmers in Table 3 below. The sample is 65% male, with an average age of about 42, and with only 3 years of formal education. The average unit of agricultural production (*Unité de Production Agricole*, or UPA) is approximately 18 people including 8 children, 4 students and one person over 70 years old. A UPA is an agricultural production unit made up of farmers, mostly members of the same family group, whether living or not in the same household (Diallo 2011). The average land holdings per UPA are 9 ha, 42 km from a main road, and 18 km from a market. They receive on average 5.5 extension visits and 86% of the sample belong to an agricultural association. The UPA size is significantly larger in Bougouni than the other locations, and the average landholding is largest in Dioila.

**Table 3:** Summary statistics of farmers in sample.

Variable	Bougoni		Dioila		Tominian		Total	
	Mean	Std.	Mean	Std.	Mean	Std.	Mean	Std.
Gender of respondent (male = 1)	0.62	0.49	0.73	0.45	0.61	0.49	0.65	0.48
Age of respondent (years)	43.86	13.85	41.45	12.24	40.52	13.05	41.92	13.09
Education (years)	3.06	1.42	3.07	1.44	2.97	1.42	3.03	1.42
Number of people in UPA	24.70	17.07	17.72	10.81	13.31	9.18	18.50	13.56
Children under 16 in UPA	10.36	7.56	8.56	6.26	6.24	3.56	8.36	6.22
Students in UPA	5.27	4.14	4.19	3.17	2.85	2.45	4.09	3.45
Older than 70 in UPA	0.76	0.91	1.41	6.77	0.64	0.87	0.93	3.98
UPA landholdings (ha)	9.94	7.42	10.41	6.83	7.43	4.32	9.27	6.46
Personally farmed (ha)	1.74	3.32	1.96	4.07	2.06	3.53	1.93	3.65
Years farming this plot	10.04	10.45	7.45	13.63	11.27	8.85	9.19	11.71
Distance to road (km)	7.57	12.31	87.46	185.85	31.23	116.79	42.34	131.33
Distance to market (km)	28.78	96.44	5.20	2.88	22.35	57.68	18.70	65.23
Extension visits	8.48	8.52	6.00	7.47	2.07	2.59	5.50	7.18
Association membership	0.81	0.67	0.76	0.45	0.49	0.50	0.68	0.56
N	104		106		108		318	

We also compare the differences in income and assets between the various sites. Participants in Bougouni have the highest non-agricultural income of the three villages while Tominian has the least opportunities for off farm income. The majority of income in Dioïla comes from small business activities. Participants in Dioïla take more loans than participants in the other villages on average, particularly in Tominian. On average Bougouni appears to be a wealthier community than Dioïla and Tominian and have more agricultural and non-agricultural assets. Large ruminants (cattle) are more common in Bougouni and least common in Tominian but small ruminants (sheep and goats) and pigs are most common in Tominian.

We asked farmers a series of questions related to sorghum cultivation and use (see Table 4). In our sample, average rainfall and percentage of farmers growing sorghum is inversely related— the lower the rainfall the higher the percentage of farmers producing sorghum. However, the largest mean land area planted to sorghum was in Dioïla (3.69 ha). There was wide variation in reported yield per hectare with the highest figures in the highest rainfall areas but the standard deviations were large and so these figures are not statistically different from one another. The total quantity consumed and sold was higher in the higher rainfall areas but the percent of the harvest consumed was smaller when more sorghum was harvested (86%, 94%, and 95% in Bougoni, Dioïla, and Tominian respectively). There is a relatively high level of intercropping with sorghum (about 50%) with the most in Dioïla and least in Tominian. The vast majority of the sorghum that was intercropped was intercropped with cowpea (89%).

**Table 4:** Sorghum production and mean descriptive statistics for sorghum production.

	HH/UPAs producing sorghum	Area (Ha^−1^)	Quantity harvested (kg/Ha^−1^)	Quantity consumed (kg/Ha^−1^)	Quantity sold (kg/Ha^−1^)	Inter-cropping
Bougouni	59%	1.34	836 (1018)	717 (979)	649 (824)	51%
Dioila	87%	3.69	715 (401)	669 (407)	288 (443)	68%
Tominian	90%	2.29	519 (521)	495 (482)	303 (275)	22%

Note: standard deviations are in parenthesis.

It is common that women in Mali will manage land separate from the main agricultural fields, producing small amounts of cereal crops for household consumption or sale. These cereals are often intercropped with women's primary crops, such as cowpea or groundnut. Since males head the UPA, when we interviewed them we asked them to report all of the sorghum they managed under the UPA while women were asked only to report the land that they managed personally. In some UPAs, there was sorghum cultivation taking place that was not managed by the women and of which they were not aware, so the figures presented above roughly illustrate the differences between the three sites but may not be precise estimates of total sorghum production. This is a function of the polygamous household structure and a major hurdle to collecting “household” level production data in Mali. Since we are interested in factors that impact individual decisions regarding sorghum production we thought it was more appropriate to ask respondents only about the land that they personally manage rather than interview married husband and wife pairs.

Sixty-three percent of farmers are aware of the concept of perennial sorghum but only 1% of the sample had tried it. Only 17% of the sample has tried “improved” varieties. Eighty-nine percent of the sample use sorghum residue as forage, 60% collect the residue and transport it to the animals, while 40% leave the residue in the field. Almost all of the farmers sampled (92%) source seed from their own production. Anecdotally, women do not have access to draft power when they needed it because the men use it for the main fields. However, in our sample, 80% of women reported having access to draft animals (and 90% of men) with slightly lower reporting not having access to draft animals when they needed it (78% of women and 85% of men).

### 4.2 Preferences for attributes of perennial sorghum

Estimations of preferences for perennial sorghum are presented in Table 5. The first column displays the results of a RPL, column two includes the same estimation with gender interactions, and the third column estimates the same model in WTP-space using sorghum yield as the scale factor. Differences in preferences by gender are estimated by interacting the gender of the respondent with the choice attributes.

**Table 5:** Random parameters logit model for perennial sorghum.

Variable	RPL		RPL w/ gender interactions		WTP-space w/ gender interactions	
	Coefficient	Error	Coefficient	Error	Coefficient	Error
*Random parameter means*						
Seed requirements	0.037^⁎⁎⁎^	0.008	0.036^⁎⁎⁎^	0.009	0.512^⁎⁎⁎^	0.098
Labor requirements	−0.107	0.096	−0.203^⁎^	0.106	−0.732	1.273
Forage	0.314^⁎⁎⁎^	0.088	0.288^⁎⁎⁎^	0.096	3.798^⁎⁎⁎^	1.035
Soil improvement	1.369^⁎⁎⁎^	0.139	1.149^⁎⁎⁎^	0.114	12.84^⁎⁎⁎^	1.034
Yield	0.103^⁎⁎⁎^	0.009	0.089^⁎⁎⁎^	0.007		

*Non-random parameter means*						
Opt out dummy	−4.719^⁎⁎⁎^	0.395	−4.113^⁎⁎⁎^	0.286	−5.203^⁎⁎⁎^	0.367
Female ∗ seed requirements			−0.007	0.013	−0.014	0.014
Female ∗ labor requirements			0.304^⁎^	0.180	0.175	0.186
Female ∗ forage			−0.013	0.154	−0.115	0.164
Female ∗ soil improvement			0.063	0.162	0.074	0.185
Female ∗ yield			−0.001	0.006	0.000	0.009

*Random parameter standard deviations*						
Seed requirements	0.081^⁎⁎⁎^	0.011	0.070^⁎⁎⁎^	0.010	0.593^⁎⁎⁎^	0.100
Labor requirements	0.958^⁎⁎⁎^	0.172	0.894^⁎⁎⁎^	0.155	8.467^⁎⁎⁎^	1.327
Forage	0.563^⁎⁎⁎^	0.195	0.524^⁎⁎⁎^	0.178	4.048^⁎^	2.104
Soil improvement	0.658^⁎⁎⁎^	0.176	0.216	0.287	4.044^⁎⁎^	1.962
Yield	0.033^⁎⁎⁎^	0.006	0.026^⁎⁎⁎^	0.006		
N	1272		1272		1272	
K	11		16		16	
Log-likelihood	−877.81		−876.84		−857.13	
Pseudo R^2^	0.372		0.373		0.387	
AIC	1777.6		1785.7		1746.3	

Note: ^⁎⁎⁎^ indicates significance at the 1% level, ^⁎⁎^ indicates significance at the 5% level, and ^⁎^ indicates significance at the 10% level, respectively. The sorghum yield coefficient estimate in WTP-space model is omitted since this is the scale factor.

We use the yield variable to calculate the marginal rate of substitution, and this is framed as a percentage reduction in the level of a respondent's sorghum harvest from the previous growing season (−60%, −40%, −20%, the same). A positive valuation of yield can be understood as how much of a yield reduction a participant would be willing to tradeoff (or accept) to have higher levels of an attribute (e.g., soil improvement). Since seed requirements are also framed as a reduction in level (average, 15% less or 30% less), a positive sign on this variable indicates a positive valuation of a reduction in seed required. Labor requirements, forage biomass, and soil improvement are all binary variables so a positive sign would indicate the difference in valuation from the low to high level. Coefficients in the WTP-space model should be interpreted as tradeoff values relative to other attributes in terms of sorghum yield. Note that the coefficient listed for yield in WTP-space is the associated preference space estimate.

The value of the coefficient on seed requirements is small, about 0.5 CFA (or about $0.005) per 1% of sorghum yield, suggesting that this attribute is a minor concern relative to the other attributes involved in the choice of the sorghum variety. Labor requirements are negative but not significant, indicating that labor requirements are not necessarily an important determinant of the choice between an annual and perennial sorghum variety overall. The random parameter standard deviation on labor is significant and relatively large however, indicating that there is heterogeneity in labor valuation. In the RPL with interactions model the value of labor to women is positive and marginally significant, based on the coefficient of the interaction between the gender and labor variables, indicating that women are less concerned than men about labor savings. This effect however washes out in the WTP model when coefficients are scaled.

As forage increases from low to high there is a positive and significant valuation but the random parameter estimate for this is not significant suggesting that this is not important to all farmers. The tradeoff value for soil improvement indicates that this attribute is roughly three times more important to farmers than forage/biomass production. In the RPL model yield is positive and significant as is the random parameter standard deviation and there is some heterogeneity in yield valuation. The opt out dummy is negative and very large and significant for perennial sorghum, indicating that many respondents prefer traditional sorghum over perennial.

### 4.3 Preferences for attributes of hybrid sorghum

Model results of preferences for hybrid sorghum are presented in Table 6. The same three models are presented: an RPL choice model, an RPL model with gender interactions, and a model with gender interactions estimated in WTP space. One important difference in the way the choice sets are constructed is that the hybrid sorghum yield variable is framed as an increase in yield compared to traditional sorghum whereas perennial was framed as a yield reduction. In the perennial model a positive valuation of yield can be interpreted as how much of a yield reduction a participant would be willing to accept while the interpretation of yield in the hybrid sorghum model is how much the respondent values a percentage increase in yield. Also in the perennial model the seed requirements variable is framed as a percentage of seed required (since less seed is required with perennial but it is not clear exactly how much) whereas in the hybrid model we specifically capture the seeding rate difference using experimental data (hybrid sorghum has a higher germination rate and so only one half the amount of seed is required).

**Table 6:** Random parameters logit model for hybrid sorghum.

Variable	RPL		RPL w/ gender interactions		WTP-space w/ gender interactions	
	Coefficient	Error	Coefficient	Error	Coefficient	Error
*Parameter means*						
Seed requirements	−0.007	0.034	−0.031	0.044	−0.809	0.705
Plant duration	−0.437^⁎⁎⁎^	0.064	−0.445^⁎⁎⁎^	0.080	−6.133^⁎⁎⁎^	1.083
Ability to reuse	2.059^⁎⁎⁎^	0.259	2.321^⁎⁎⁎^	0.309	33.300^⁎⁎⁎^	4.171
Cost of seed	0.000	0.000	0.000	−0.280	−0.001	0.005
Yield	0.071^⁎⁎⁎^	0.007	0.073^⁎⁎⁎^	0.008		

*Non-random parameter means*						
Opt out dummy	1.073	0.872	1.139	0.872	3.898^⁎⁎⁎^	0.610
Female ∗ seed requirements			0.063	0.067	0.091	0.073
Female ∗ plant duration			0.004	0.121	−0.054	0.137
Female ∗ ability to reuse			−0.758^⁎⁎^	0.345	−0.354	0.435
Female ∗ cost of seed			−0.001	0.001	−0.001	0.001
Female ∗ yield			−0.004	0.008	−0.005	0.006

*Random parameter standard deviations*						
Seed requirements	0.134^⁎^	0.076	0.132^⁎^	0.079	1.468	1.710
Plant duration	0.017	0.133	0.025	0.133	0.463	15.248
Ability to reuse	1.845^⁎⁎⁎^	0.274	1.829^⁎⁎⁎^	0.282	30.929^⁎⁎⁎^	3.934
Cost of seed	0.002^⁎⁎⁎^	0.000	0.001^⁎⁎⁎^	0.000	0.033^⁎⁎⁎^	0.006
Yield	0.041^⁎⁎⁎^	0.006	0.041^⁎⁎⁎^	0.006		
N	1272		1272		1272	
No. of parameters	11		16		16	
Log-likelihood	−744.984		−740.203		−765.51482	
Pseudo R^2^	0.466		0.470		0.451769	
AIC	1512		1512.4		1563	

Note: ^⁎⁎⁎^ indicates significance at the 1% level, ^⁎⁎^ indicates significance at the 5% level, and ^⁎^ indicates significance at the 10% level, respectively. The sorghum yield coefficient estimate in WTP-space model is omitted since this is the scale factor.

The respondent is presented with a choice that is not explicitly hybrid versus non-hybrid sorghum but rather two choices that differ in attributes that characterize the main differences between hybridized and non-hybridized sorghum crops. Seed requirements are not an important component of the tradeoff to farmers and there is little variation in this preference. Sorghum maturity is important, indicated by the significant tradeoff value and there is a significant and homogenous preference for shorter duration sorghum. The ability to reuse the seed is the most important attribute to participants in the choice, approximately 6 times more important than earlier maturity. On average women put slightly less emphasis on the ability to reuse seed than their male counterparts (seen in the RPL model), but there is significant variation within this group and this effect washes out in the WTP-space model.

Sorghum yield is positive, relatively large and also significant indicating the importance of yield for hybrid seeds. However, there is heterogeneity in the valuation of sorghum yield. The coefficient on the opt out dummy in the WTP-space model is positive and significant, indicating an overall preference for hybrids sorghum over traditional sorghum.

## 5 Discussion

Perennial and hybrid crops present fundamentally different technological options to farmers and there is evidence of demand for attributes of each technology and evidence that they might fit into men and women's cropping systems differently. The two statistics that are the most prominent from these estimations is that the most important attribute of perennial sorghum is soil improvement and the most important attribute of hybrid sorghum is whether the seeds can be reused. We address each of these attributes in more detail.

Farmers' main interest appears to be improvement in soil quality from the perennial plant, which is consistent with pigeon pea farmers in Malawi (Waldman et al. 2017). Soil improvement is the most important determinant of farmer choice, approximately three to four times as important as biomass production. The benefits of perenniality in terms of reduced seed and labor requirements appear to be negligible to farmers. While previous research has found that perennial crops improve soil (Culman et al. 2013) and soil structure (Kell 2011), there is no evidence of direct soil fertility improvement from perennials since the improvement happens over such a long time horizon. Developing perennial sorghum varieties that are primarily soil enhancing with more extensive root systems and higher above ground biomass is an area where further breeding efforts could increase adoption by farmers. Efforts to improve soil fertility should aim to keep yields within relatively similar to annual sorghum yields in subsequent years of production to remain appealing to farmers. However, since labor and seed requirements are not as important to farmers and distinguishing attributes of a perennial crop there will likely be extensive behavioral barriers to adoption of perennial sorghum in Mali.

Hybrid crops will likely face resistance because of the long tradition of saving and reusing seeds among traditional cultures in Mali and elsewhere. The traditional practice of hand selecting varieties with desirable attributes and replanting them in subsequent seasons (and seed recycling) is ingrained in farmers. Hand selecting crops over time allows them to adapt to local variation in climate and to localized pest and disease outbreaks. Manual seed selection and reusing seed gives farmers control over their own seed supply and buffers them fluctuating seed prices. There is also a great deal of misinformation associated with hybrid crops, and associations with genetic modification of crops that carry a negative stigma in rural parts of Mali (as well as across Africa). The process of hybridization is thus a barrier to the proliferation of the technology. However, because of the yield advantages from hybrids some farmers are likely to incorporate hybrid sorghum varieties into their existing cropping systems alongside traditional or even perennial varieties.

The duration of the plant is important to farmers and there is a strong and homogenous preference for shorter duration crops. This appears to extend to perennial sorghum which had high and significant opt out values among farmers. Seed requirements and labor requirements are the core of the differences between an annual and a sorghum crop and both of these attributes were valued very little by farmers. This suggests that unless perennial crops can make significant ecosystem service contributions, particularly in terms of soil improvements they are not likely to be adapted simply by the different management requirements of the cropping system.

Anecdotally, sorghum is not traditionally the woman's domain in Mali, although some women grow small plots of sorghum for income generation. Women might not have much decision-making power over the land or the inputs used and so increasing forage and soil through perennial crops could be more appealing to female farmers. Given the prevalence of polygamous households in Mali, women generally have less access to cash and assets and often farm smaller plots of land with fewer resources. For some women, labor may not be a limiting constraint, likely because the plots of land they are farming are so small that they can personally supply enough labor. However, for women farmers with a labor shortage a perennial crop might be an advantage to them since fewer plowings are required. Regardless there is heterogeneous demand for labor savings, so a perennial crop is potentially an important technology for resource-poor women and may provide an important incentive for adoption of sorghum.

## 6 Conclusions

Attributes of both perennial and hybrid varieties are positively valued by farmers and the technologies could be considered as complementary and part of a risk diversification strategy for smallholder farmers in Africa. There are constraints to widespread adoption of either technology, which highlights the importance of understanding the tradeoffs between agricultural ecosystem services and climate change adaptation. These findings have important policy implications for adaptation to climate change. This is particularly true of perennial grain crops that have not been well developed in the region or on the continent. Hybrids also face numerous behavioral barriers and in particular the hybridization process runs contrary to cultural drivers of seed choice, specifically the prevalence of seed reuse or recycling. To be consistent with farmers' preferences, breeding efforts in Africa should continue to focus on crop diversity and diversification into crops other than maize to provide risk averse farmers with more options which fit their agro climatic conditions and individual farming systems.

Breeders should be mindful of the two major findings of these experiments. The first is that perennial crops have little appeal to farmers unless they can provide substantial ecological benefits. The ability to grow in drier climates is a potentially very powerful aspect of perennial crops in a future characterized by rising temperatures and greater variability in precipitation. Increased forage biomass and improved soil fertility from perennial crops is promising to farmers, but only at a relatively small yield reduction. The second major finding is that acceptability of hybrid crops is largely hampered by the ability to recycle the seeds. Farmers value shorter duration crops, but this is far outweighed by their aversion to seeds they cannot recycle.

Developing perennial lines of sorghum that can survive the dry seasons in Mali could be a very important technology for climate change adaptation. Enhancing and documenting the ecosystem services and other ancillary benefits provided by perennial crops will be key to farmer adoption. Early maturing hybrids are also a promising technology to reduce exposure to climate risk and adapt to changing climatic conditions. Working with farmers to produce hybrid seed at the local level is one potential way to both create a local market for hybrids and to enable farmers to create a sustainable and appealing seed system that involves hybrids.

## Footnotes

1 Including Institut d'Economie Rurale and the International Crops Research Institute for the Semi-Arid Tropics (ICRISAT) both based in Bamako.

2 1 USD is equivalent to approximately 586 West African CFA or Communauté Financière Africaine (March 2014).
